# The cost-effectiveness of an eradication programme in the end game: Evidence from guinea worm disease

**DOI:** 10.1371/journal.pntd.0005922

**Published:** 2017-10-05

**Authors:** Christopher Fitzpatrick, Dieudonné P. Sankara, Junerlyn Farah Agua, Lakshmi Jonnalagedda, Filippo Rumi, Adam Weiss, Matthew Braden, Ernesto Ruiz-Tiben, Nicole Kruse, Kate Braband, Gautam Biswas

**Affiliations:** 1 Department of Control of Neglected Tropical Diseases, World Health Organization, Geneva, Switzerland; 2 Guinea Worm Eradication Program, The Carter Center, Atlanta, Georgia, United States of America; 3 Development Office, The Carter Center, Atlanta, Georgia, United States of America; RTI International, UNITED REPUBLIC OF TANZANIA

## Abstract

**Background:**

Of the three diseases targeted for eradication by WHO, two are so-called Neglected Tropical Diseases (NTDs)–guinea worm disease (GWD) and yaws. The Guinea Worm Eradication Programme (GWEP) is in its final stages, with only 25 reported in 2016. However, global eradication still requires certification by WHO of the absence of transmission in all countries. We analyze the cost-effectiveness of the GWEP in the end game, when the number of cases is lower and the cost per case is higher than at any other time. Ours is the first economic evaluation of the GWEP since a World Bank study in 1997.

**Methods:**

Using data from the GWEP, we estimate the cost of the implementation, pre-certification and certification stages. We model cost-effectiveness in the period 1986–2030. We compare the GWEP to two alternative scenarios: doing nothing (no intervention since 1986) and control (only surveillance and outbreak response during 2016–2030). We report the cost per case averted, cost per disability adjusted life year (DALY) averted and cost per at-risk life year averted. We assess cost-effectiveness against a threshold of about one half GDP per capita (less than US$ 500 in low income countries). All costs are expressed in US$ of 2015.

**Results:**

The GWEP cost an estimated US$ 11 (95% uncertainty interval, 4.70–12.49) per case averted in the period 1986–2030. The pre-certification and certification phases can cost as much as US$ 0.0041 and US$ 0.0015 per capita per year. The cost per DALY averted by the GWEP relative to doing nothing is estimated at US$ 222 (118–372) in 1986–2030. The GWEP is probably more cost-effective than control by the year 2030. The GWEP is certainly more cost-effective than control if willingness to pay for one year of life lived without the risk of GWD exceeds US$ 0.10.

**Discussion:**

Even if economic costs are two times as high as the financial costs estimated for the period to 2020, the GWEP will still be cost-effective relative to doing nothing. Whether the GWEP turns out to be the most cost-effective alternative in the period beyond 2015 depends on the time horizon. When framed in terms of the number of years of life lived without the risk of GWD, a case can be made more easily for finishing the end game, including certification of the absence of transmission.

## Author summary

Of the three diseases targeted for eradication by WHO, two are Neglected Tropical Diseases (NTDs)–guinea worm disease and yaws. The decision to pursue eradication of these diseases was based, in part, on economic arguments made at a time when case numbers were high. There is, in fact, little published evidence of the cost and cost-effectiveness of an eradication programme in the end game, when the number of cases is lower and the cost per case is higher than at any other time. The Guinea Worm Eradication Programme (GWEP) is in its final stages, with only 25 cases reported in 2016. Ours is the first economic evaluation of the GWEP since a World Bank study in 1997. For the first time for any eradication programme, we document the full cost of certifying the absence of transmission in all countries with a history of the disease. We analyze the cost-effectiveness of the GWEP to find that it remains highly cost-effective in spite of high costs in the end game. These results will be of interest to funders of guinea worm disease and yaws eradication, but also to policy makers considering eradication of other NTDs.

## Introduction

Eradication is the “permanent reduction to zero of the worldwide incidence of an infection caused by a specific agent as a result of deliberate efforts; intervention measures are no longer needed”.[1] Only one human disease has ever been eradicated. The eradication of smallpox (formally declared in 1980) is estimated to have avoided 1.5 million deaths per year in developing countries and led to a benefit of about US$ 1070 million per year globally. The economic benefit to industrialised countries of avoided vaccination costs alone amounts to about US$ 350 million per year.[2]

Of the three diseases currently targeted for eradication by the World Health Organization (WHO), two are so-called Neglected Tropical Diseases (NTDs)–guinea worm disease (GWD) and yaws. The economic benefit of GWD eradication will be smaller in absolute terms than that of smallpox, because GWD does not affect the developed world. Nonetheless, GWD eradication will be a major victory for public health. It will be the first parasitic disease to be eradicated (smallpox is a virus) and the first disease to be eradicated without the use of a vaccine or medicine.[3] Progress towards eradication has already been a major victory for the “forgotten people of forgotten places” who no longer suffer from GWD and its effects on such communities, despite the fact that their standards of living have not improved very much, if at all, since the eradication campaign began.

The Guinea Worm Eradication Programme (GWEP) is in its final stages, with only 22 cases reported in 2015 and 25 cases in 2016. However, the target date for eradication has been pushed back a number of times (first 1995, then 2009, then 2015). The inability of the campaign to meet the first two arguably over-ambitious target dates set by the World Health Assembly was due to a lack of funding to support national eradication efforts. Failure to meet the 2015 target is attributed primarily to insecurity (especially armed conflict) in some of the remaining endemic countries, as well as unexpected new modalities of transmission (through dogs) in Chad.[4]

Eradication requires formal certification by WHO of the absence of transmission in all countries including those with a history of endemicity. WHO established the International Commission for Certification of Dracunculiasis Eradication (ICCDE) in 1995, “to evaluate the status of countries applying for certification of dracunculiasis eradication and to recommend whether a particular country should be certified as free of transmission.”[5] A country endemic for GWD “reporting zero indigenous cases over a complete calendar year is deemed to have prevented transmission of guinea-worm disease and is classified in a precertification stage”.

To be declared free of GWD, “a country that has stopped transmission of the disease must have reported zero indigenous cases through active surveillance for at least three calendar years.”[6] Prerequisites are that:

1. Surveillance activities of an adequate standard have been undertaken for at least three years since the last reported indigenous case.
2. In the event of an imported case, a full investigation has been performed to confirm the endemic area of origin; full case containment activities have been undertaken.
3. A register of suspected cases has been maintained; their movements and activities have been documented and all sources of potentially contaminated drinking water have been identified.

A national report documents “all actions taken from the beginning of the programme, including the three-year pre-certification period, to interrupt transmission and confirm zero occurrences of guinea-worm disease cases.” An International Certification Team (ICT) then visits the country to verify the information in the national report: “During its visit, ICT assesses the adequacy of the surveillance system and reviews records of investigations for rumoured cases and subsequent actions taken.”[6]

The last formal economic evaluation of the GWEP was undertaken in 1997 by the World Bank (WB), when the number of cases was estimated at about 330 000 cases (with 152 185 cases being reported).[7] At that time, the cost of the programme (1987–1998) was estimated at US$ 87.46 million (nominal prices, unadjusted for purchasing power) or 1987 US$ 68.46 million (constant prices, adjusted for purchasing power). That study assumed that in the absence of the GWEP, the incidence of cases would have remained at the level of 1986 (prior to initiation of activities) or 2.25 million cases per year. It estimated that by 1998, 13 million cases would have been prevented by the GWEP, at a cost of about US$ 5 per case averted (at 1987 prices) and an economic rate of return (ERR) of 29%.

The case for investment in 2016, when there were 25 cases, is not the same as in 1997, when there were still tens of thousands of cases of GWD. The WB study considered a project horizon up to the year 1998 only, while recognizing that “the longer it takes for eradication efforts to be successful, the lower are the projected economic returns”.[7] By 2004, about US$ 125 million had been spent and another US$ 53.5 million committed through to 2010.[8] Costs have therefore increased to at least double the WB estimate.

In this paper, we update the investment case for the GWEP. We analyse the cost-effectiveness of the GWEP in the end game, when cases are low, the cost per case is high, and much of the cost is for pre-certification and certification rather than implementation. We compare the GWEP to two alternative scenarios: doing nothing (no intervention since 1986) and control (surveillance and outbreak response only in 2016–2030, without certification of global eradication).

### Guinea worm disease (GWD)

GWD is caused by the parasitic worm *Dracunculus medinensis*, which infects people who drink water from stagnant sources containing tiny copepods (“water fleas”) harbouring microscopic infective larvae. Approximately one year after infection, adult female worms measuring up to one metre emerge painfully through a person’s skin. When the wound is cooled in water, the worm releases hundreds of thousands of larvae, contaminating the source and continuing the cycle of the disease. The emerging worm makes it difficult for hosts to walk, care for themselves, grow food, work or attend school.[9–11] Secondary bacterial infections usually ensue and exacerbate pain and prolong recovery time, and may lead to permanent disability. Although rare today, death as a result of tetanus was not an unusual event during the early years of the GWEP.

Endemic transmission of GWD is a consequence of extreme poverty in remote and marginalized communities of sub-Saharan Africa. Standards of living in these communities have not changed much or at all since the 1980s, when the GWEP began. Hospitals and clinics are often absent or kilometres away, hindering access to modern medical care. The absence of a known cure is another barrier to seeking modern medical care. There is no drug to cure GWD or vaccine to prevent it, and humans do not develop immunity to the disease. However, disease transmission can be prevented.

### The Guinea Worm Eradication Programme (GWEP)

**Fig 1:**
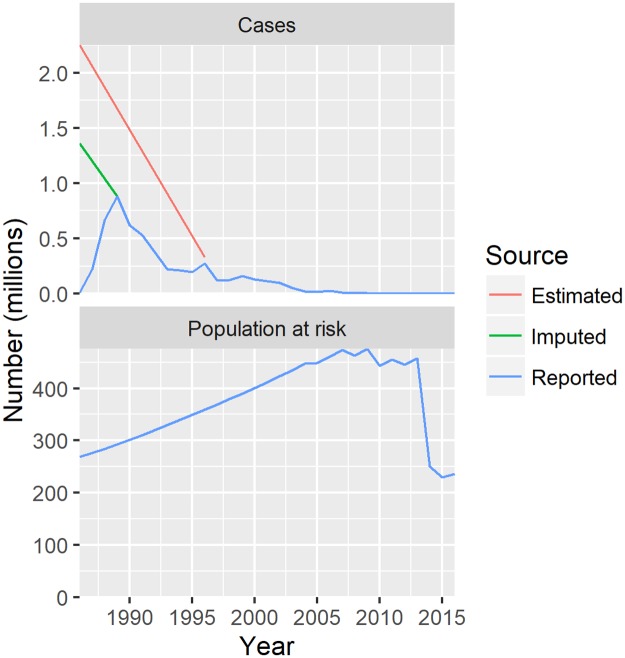
Number of cases and population at risk, 1986–2016. Reported values for 1986–2016 are as per WHO data; imputed value for 1986 is the sum of the maximum values for each country in the period 1986–2016; estimated values for 1986 and 1996 are as per World Bank study (1997).

GWD eradication efforts started in the 1980s, just after the successful eradication of smallpox in 1979. In 1986, the first World Health Assembly (WHA) resolution on GWD called on affected Member States to “establish as quickly as possible, within the context of primary health care, plans of action for eliminating dracunculiasis, giving high priority to endemic areas in providing safe sources of drinking water.”[12] WHO, The Carter Center (TCC), the United Nations International Children's Emergency Fund (UNICEF), and United States Centers for Disease Control and Prevention (CDC) became the lead organizations of a global programme to eradicate GWD. Working together with the ministries of health in endemic countries and a coalition of partner organizations, GWD has been reduced more than 99.9 percent. Fig 1 shows the dramatic drop from an estimated 3.5 million cases occurring annually in 21 countries in 1986 to 25 cases reported in four countries in 2016.

As there is no medical treatment for GWD, the strategy for eradication relies on the identification of all villages with endemic transmission of GWD and interrupting transmission in each. National GWEPs, with support from partners, interrupt transmission of GWD by creating and sustaining networks that permit: 1) community-based education of residents about the disease and what they can do to prevent infected residents from contaminating sources of drinking water via prompt detection and containment of cases; 2) filtration of all drinking water through cloth filters and pipe filters; and 3) treatment of contaminated stagnant water sources with ABATE larvicide. In addition, since its inception, national GWEP programs have advocated with water sector organizations for the provision of safe sources of drinking water to affected communities.

The current role of the ICCDE and WHO within the GWEP includes verification of the absence of transmission via assessment of the surveillance quality during the three year pre-certification period. Since its establishment in 1995 to the end of 2016, the ICCDE has met eleven times.[13] It has certified 198 countries, territories and areas (belonging to 186 WHO Member States) as free of GWD. The latest country to attain this status in January 2015 was Ghana. The population living in endemic countries has been halved since 2013, with certification of these countries (Fig 1).

An additional eight countries await certification: four endemic countries (Chad, Ethiopia, Mali and South Sudan); two countries in the pre-certification phase (Kenya and Sudan); and two which have not reported any recent history of the disease (Angola and the Democratic Republic of Congo).To achieve global certification of GWD eradication, “WHO must formally certify every individual country even if no transmission has ever been recorded in that particular country.”[5]

WHO is providing financial and technical assistance in all eight countries yet to be certified, including for surveillance at cross border areas and in refugee camps in the four endemic countries. It has full responsibility in supporting pre-certification activities in Kenya and Sudan, as well as in supporting full verification of Angola and the Democratic Republic of Congo for certification. In addition, it provides assistance for surveillance in at least ten certified countries at risk of disease re-introduction.

TCC has responsibility for assisting national GWEPs to interrupt transmission. From mid-2015, TCC has also been responsible for supporting the four remaining endemic countries to prepare for certification up to 36 months after interruption of transmission. Activities include providing financial and technical assistance for the surveillance system, investigating rumours of possible cases, responding to outbreaks, establishing a reward system for reporting cases, developing capacity for rigorous investigation and reporting of cases, and providing monthly reports about these investigations.

Other partners, namely UNICEF and Water Aid, have focussed on provision of safe, clean water (in particular, by providing boreholes).

## Methods

### Data collection

For the years 1986–1996, financial costs were retained from the WB study. For the period after 1996, we extracted financial costs from the records of TCC and WHO, including the ICCDE.

TCC’s financial costs were extracted for the years 2008–2015, including the cost of implementation activities per country per year. Data were available from the 10 countries in which activities were still ongoing in the period 2008–2015, namely: Burkina Faso, Chad, Ethiopia, Ghana, South Sudan, Mali, Nigeria, Niger, Sudan, and Uganda.

TCC costs include the financial cost of in-kind donations, as reported to TCC by their donors. These include the production cost of cloth for filters from E.I. DuPont Corporation and chemical larvicide (ABATE) from BASF (formerly American Home Products).[8]

WHO’s financial costs in the years 2000–2007 and 2009–2014 were available by year and categorised by type (staff and other personnel costs; contractual services; property and equipment; general operating expenses; supplies, commodities, materials; transfers and grants to counterparts and travel). Whatever assets Ministries of Health deployed for the project were largely purchased by WHO during this timeframe and are included here. Data included disbursements to 30 countries and 27 organisations.

We also extracted demographic and epidemiological data from WHO and other United Nations sources. These include the total population (since 1986, with projections to 2030), and the number of cases (1986–2015). These data are publicly available for all countries endemic for GWD.

### Cost description

We included all available financial costs from the perspective of providers (TCC, WHO and national GWEP programmes) as well as in-kind donors. All costs were converted to US$ of 2015 (2015 US$) using the GDP deflator for the United States.[14]

We combined both TCC and WHO sources to obtain total financial costs by country and year in the period 2008–2015. We analysed unit costs by phase (implementation, pre-certification and certification) and year within each phase. We calculated the following unit costs: cost per case and cost per capita for the intervention phase; cost per capita for the pre-certification and certification phases. We extracted the median, 2.5^th^ and 97.5^th^ centile values of cost per capita across all available countries and years within a phase.

Using these phase-specific unit costs, we imputed missing costs in the years 1997–2007. Given uncertainty around these imputations, we employed probabilistic sensitivity analysis (PSA). We assumed triangular distributions, using the median and centiles as the mode and min and max, respectively. We ran 1000 iterations of all calculations and extracted the mean as well as 2.5^th^ and 97.5^th^ centile values for the uncertainty intervals.

We compare financial costs of the GWEP to the null scenario of having done nothing since 1986; the null scenario is also referred to as the zero cost scenario.

We consider also a control scenario in which in the period 2016–2030, rather than pursuing eradication, surveillance and outbreak response activities alone are maintained indefinitely at pre-certification and certification levels (i.e. at the same unit cost per capita) in endemic/pre-certified and certified countries, respectively. The assumption is that surveillance during pre-certification is the minimum required to detect recrudescence and respond to an outbreak; surveillance during certification phase is the minimum required to detect case importation and respond.

Activities undertaken to interrupt transmission (during the implementation phase) and certify countries (during the certification phase) have been described in the introduction, under “The Guinea Worm Eradication Programme (GWEP)”. District-level, risk-based surveillance activities are detailed in Supporting Information S1 Table, for endemic districts (typical of the implementation phase), high risk districts (pre-certification phase) and normal risk districts (certification phase).

### Markov modelling

We developed a compartmental (Markov) model, depicted in Supporting Information S2 Figure. Transition probabilities from one state to another are determined by epidemiological parameters, with distributions for PSA (Supporting Information S3 Table). We converted rates and durations into probabilities. We converted all probabilities in weekly cycle probabilities.

The population at risk moves to or through one of the following possible states: asymptomatic infections, symptomatic but uncomplicated cases, complicated cases, cases with permanent disability, and death (the terminal state). Uncomplicated cases experience disfigurement, with pain and itch, for at least 2–4 weeks, as the worm emerges. About half become complicated cases, with secondary bacterial infections, abscesses, arthritis, contracture of joints and severe disability lasting up to another 16 weeks. In fact, pain can persist as much as 12–18 months after the emergence of worms in about a quarter of cases.[15] The case fatality rate is about 0.1%. Permanent disability (e.g. “locked” knees or other joints) occurs in about 0.5% of all cases.

In order to keep the model tractable, we conservatively assume that individuals in the permanent disability state are not re-infected or at least that there is no additional disability weight associated with symptomatic infection when the individual is already in a state of permanent disability.

Start values for the number of new (incident) cases are based on, as a minimum, the highest number of reported cases since 1986 (1.3 million cases) and, as a maximum, the 2.25 million cases cited in the WB study. We allowed the model to run over a period of 100 years or 5200 weeks to populate the states of the model; the observations of this “burn in” period were discarded. The model was then run for a period of 45 years or 2340 weeks, coinciding with the period 1986–2030. In reality the benefits of eradication would extend well beyond 2030.

The model was run twice, to estimate health effects 1) under the GWEP, and 2) under the null scenario (no intervention). The epidemiological parameters that differ between the two are: 1) the reproduction number, described below; and 2) the probability of complications (which indirectly impacts also on the probability of permanent disability). Complications are reduced from 50–76% of cases under the null, to 25–50% of cases under the GWEP.

Health effects under the control scenario are the same as under the GWEP until 2015; given the lack of data on what would happen in the absence of implementation activities after 2015, we assumed that surveillance and outbreak response activities succeed in maintaining incidence at 2015 levels, but never achieving eradication.

For the null scenario, we conservatively assumed no increase in the number of cases over time (an average of 0.985–0.999 secondary cases generated by an index case over the course of the infectious period). For the GWEP, we took monthly data on the number of cases, available from 1999 to 2015 (a period of roughly exponential decrease). We performed a panel linear model (with fixed country effects) of the logarithm of cases on the month number to obtain an average monthly rate of decrease, with 95% confidence interval. The estimated effective (or actual) reproduction number (R_n_) reflects the control efforts in place in that period. The formula is:

$$R_{n}\;=\;\Lambda^{2}\;\times \;\left(L\;\times \;D\right)\;+\;\Lambda \;\times \;\left(L\;+\;D\right)\;+\;1$$

where *L* and *D* are the average durations of the latent and infectious periods, respectively and *Λ* is the rate of decrease.[16] The above equation holds when the latent and infectious periods are assumed to follow the negative exponential distribution.[17] The combined average durations of the latent and infectious periods (L+D) is known as the serial interval or generation time—that is, the time interval between successive cases in a chain of transmission. In the case of GWD, we assume an infectious period (*D*) of 1–2 weeks and a generation time of 45–65 weeks. We used the 95% confidence interval on *Λ* and ranges for *L* and *D* to calculate a range of plausible values for *R*_*n*_.

After running the two models, we weighted calculated disability-adjusted life years (DALYs). There are no disability weights specific to GWD; we therefore referred to generic health states from the Global Burden of Disease Study 2010.[18] For GWD without complications, we used 0.188 (95% CI 0.125–0.267) based on disfigurement level 2 with itch or pain, described as: “a visible physical deformity that is sore and itchy. Other people stare and comment, which causes the person to worry. The person has trouble sleeping and concentrating.”[18] For GWD with complications, we used 0.295 (95% CI 0.196–0.409) based on gout: acute, described as: “severe pain and swelling in the leg, making it very difficult to get up and down, stand, walk, lift, and carry heavy things. The person has trouble sleeping because of the pain.” Conservatively, these disability weights were applied only to a maximum of 20 weeks, not the 12–18 months reported elsewhere.[15]

For permanent disability, the disability weight is provided in Supporting Information S3 Table.

In the absence of detailed information on the percentage of cases with infection by multiple worms (in particular, how this percentage has changed under the GWEP), we have conservatively assumed that the uncertainty intervals on the duration of disease and on the disability weight capture also infection by multiple worms.

Again, we ran 1000 iterations of all calculations and extracted the mean as well as 2.5^th^ and 97.5^th^ centile values for the uncertainty intervals.

### Cost-effectiveness analysis

We estimated the cost per life year at risk averted, cost per case averted, and cost per DALY averted in the periods 1986–1996, 1986–2020 and 1986–2030. We applied willingness-to-pay (WTP) thresholds of about one half of GDP per capita to the cost per DALY averted, or less than US$ 500 in low-income countries. One half of GDP per capita is conservative compared to traditional WTP thresholds of one to three times GDP per capita.[19,20]

Life years at risk are defined as the number of years of life lived in GWD endemic countries. The entire population of a country is assumed to remain at risk until that country is certified as free of GWD. As described above, we consider also a control scenario in which in the period 2015–2030, rather than pursuing eradication, surveillance and outbreak response activities are maintained indefinitely at pre-certification and certification levels in endemic/pre-certified and certified countries, respectively. In the control scenario, therefore, the populations of countries not yet certified in 2015 are never removed from risk.

Average cost-effectiveness ratios (ACERs) were calculated relative to the null scenario of having done nothing since 1986. There is some inconsistency in the literature about the definition of the ACER; however, the alternative of dividing total cost by total effect is not considered informative. Instead we compared the costs and effects of each scenario (eradication or control) with a single option, "do nothing".

We discounted all costs and effects by between 0 and 3% per annum, applying the same rate to both costs and effects. Using the 1000 iterations of costs and effects, we calculated the cost-effectiveness, and extracted the mean as well as 2.5th and 97.5th centile values for the uncertainty intervals.

It is worth noting here the ways in which our methods differ from that of the 1997 WB study.

The WB study calculated the cost per case averted, but not the cost per DALY averted. It relied on a human capital approach to generate cost-benefit ratios. The human capital approach places monetary weights on healthy time using market wage rates. The WB study used agricultural value-added and the assumption that a 1% increase in labour input increases agricultural output by 0.66% (output elasticity of labour); it assumed that on average 5 weeks of production time (12.5% of annual work time) is lost per case of GWD.

The human capital approach is based on a strong assumption of full employment. To be fair, the WB study addressed this issue head-on, arguing that “unemployment is not a major factor in the analysis. … The rural labor [sic] sector (on which this study exclusively focuses) primarily comprises unskilled workers (with relatively low levels of education) as well as subsistence farmers. Therefore increases in productive labor [sic] time are expected to result in the augmentation of agricultural output.”[7]

Nonetheless, the approach is problematic in informal settings where agricultural value-added is hard to measure. Moreover, it ignores the suffering of children and the elderly (those not part of the “productive age group” referred to in the WB study). More pragmatically, the approach results in measures such as ERR that cannot be readily compared across diseases priorities for which cost per DALY averted is the standard measure.

In summary, we update the cost per case averted, but do not update the ERR and focus instead on the cost per DALY averted as the primary measure of cost-effectiveness.

## Results

### Cost description

**Fig 2:**
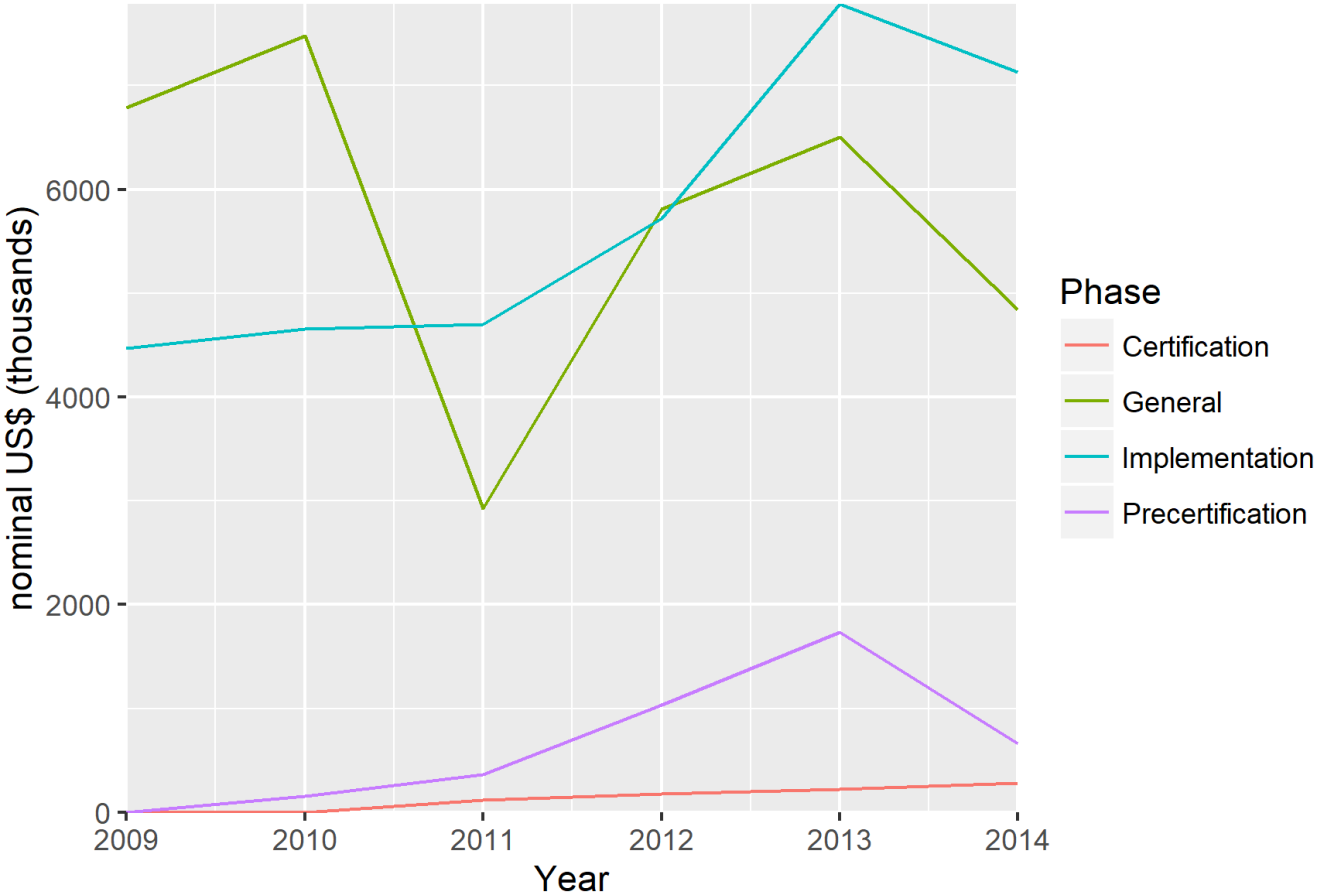
Annual financial costs of the GWEP, by country phase and general, 2009–2014. General costs refer to costs incurred by global and regional organizations for multi-country activities; costs are expressed in nominal prices (unadjusted for purchasing power).

In Fig 2, annual financial costs of the GWEP are presented, by country phase, for the period 2009–2014. Most of the cost has been for implementation. However, many costs are not country-specific. These “general costs” refer to costs incurred by global and regional organizations for multi-country activities and can therefore not be assigned to any one phase. There are also significant costs associated with the pre-certification phase. Annual pre-certification costs range from about US$ 343 000 in Côte d’Ivoire to more than US$ 1.6 million in Nigeria. Total spending is influenced by population size and land mass.

**Fig 3:**
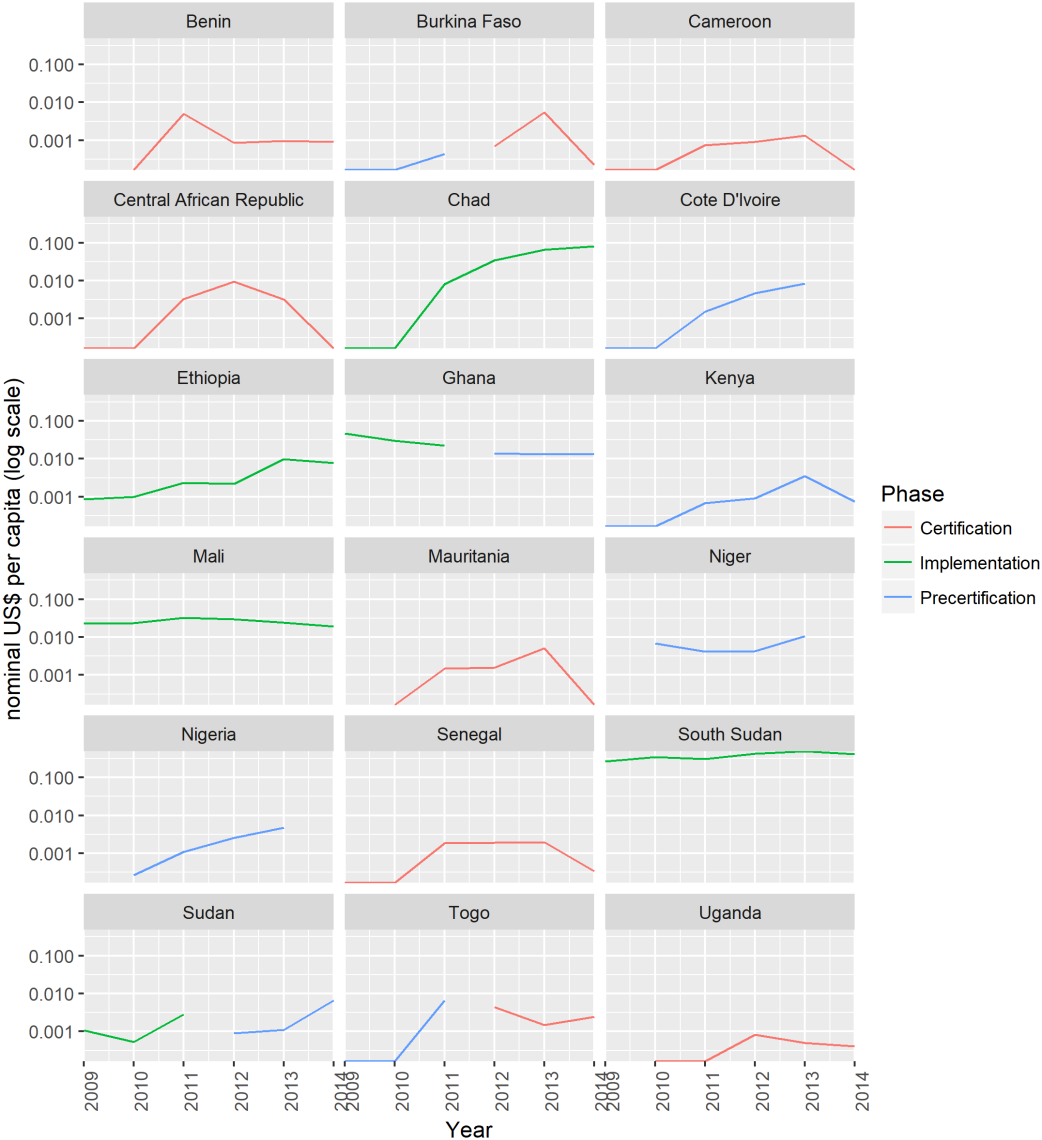
Annual financial costs per capita of the GWEP, by country, 2009–2014. Costs are expressed in nominal prices (unadjusted for purchasing power).

Fig 3 shows these annual financial costs by country, on a per capita basis (divided by the total population of that country). Note that the y-axis is on a logarithmic scale. It reveals considerable cross-country variation. Implementation costs are particularly high in South Sudan relative to other countries. Ongoing civil unrest has “periodically delayed programme implementation due to restricted access for health-care workers; programme staff undertaking active surveillance, case detection, and case containment activities; and population displacement between areas where dracunculiasis is endemic and those where it is not present.”[21] Steadily increasing costs in Chad reflect the occurrence of dog infections from 2013.[4]

Countries that moved from implementation to pre-certification (Ghana and Sudan) saw little change in costs. During pre-certification, unit costs tend to increase over time. An exception is Kenya, which has been in pre-certification for over 15 years and has had relatively low spending per capita throughout the period 2009–2014.

Countries that moved from pre-certification to certification (Burkina Faso and Togo) saw little change or a small increase in costs. Countries that have been certified do need to undertake some surveillance activities to prevent outbreaks from imported cases. Civil unrest and displacement within neighbouring endemic countries requires certified countries to heighten surveillance. However, resource requirements are less than those required in countries that are endemic or undergoing pre-certification.

Table 1 summarizes per capita costs in all phases, proving the median and 2.5^th^ and 97.5^th^ centile values used in our imputation of costs. The average cost of implementation is US$ 0.0176 per capita per year. The average costs of pre-certification and certification are US$ 0.0041 per capita per year and US$ 0.0015 per capita per year, respectively. Certified countries on average spend 22 times and 5 times less compared to endemic countries and countries undergoing pre-certification respectively. For the implementation phase, we can compare cost per capita to the cost per case (Table 1). The range on the cost per case per year is wide, extending from US$ 533 to US$ 166 951.

**Table 1:** Unit cost per year by phase, 2015 US$, median (2.5^th^ and 97.5^th^ centiles), 2009–2014.

	Implementation	Pre-certification	Certification	General^1^
**Cost per case**	31224 (533–166951)	NA	NA	NA
**Cost per capita**	0.0225 (0.0000–0.4163)	0.0008 (0.0000–0.0138)	0.008 (0.0000–0.0054)	0.0132 (0.0061–0.0416)
Excluding South Sudan	0.0128 (0.0000–0.0709)	same	same	same
Excluding South Sudan and zeros^2^	0.0176 (0.0004–0.0717)	0.0041 (0.0004–0.0139)	0.0015 (0.0002–0.0066)	same

^1^ General costs are costs incurred by global and regional organizations for multi-country activities.

^2^ Zeros refer to countries that did not undertake any activities in a given year.

**Fig 4:**
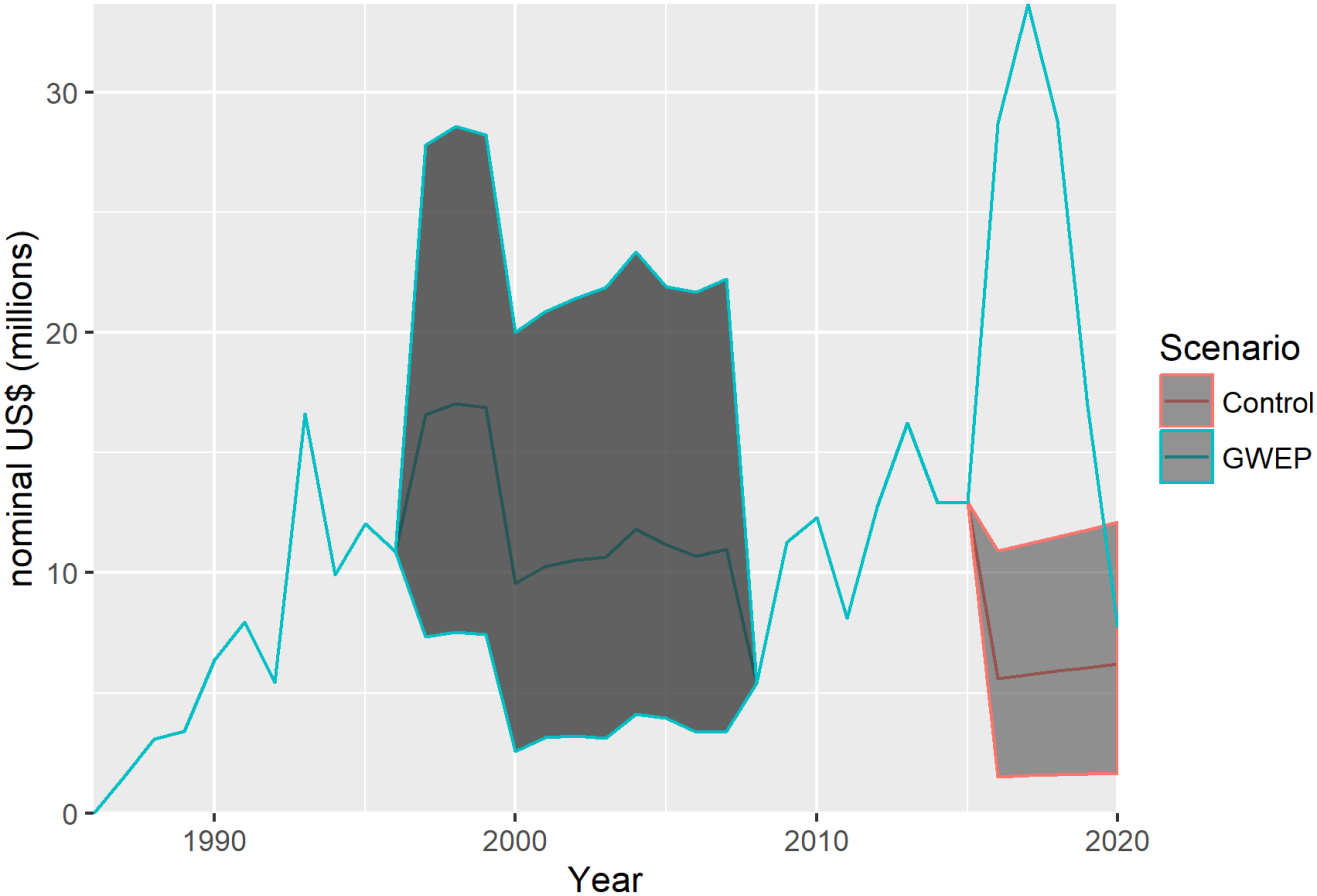
Annual financial costs of the GWEP and control scenarios, 1986–2020, best estimate and 95% uncertainty intervals. The control scenario assumes surveillance and outbreak response activities at the level of pre-certification and certification unit costs in endemic/pre-certified and certified countries, respectively, including multi-country activities; costs are expressed in nominal prices (unadjusted for purchasing power).

Fig 4 gives annual financial costs of the GWEP and control scenarios in the years 1986–2020, with reported costs in the years 1986–1996 and 2008–2015 and best estimates and 95% uncertainty intervals (grey area) in the years 1997–2007 and 2016–2020. The GWEP and control scenarios differ only in the period 2016–2020. Recall that the control scenario assumes surveillance and outbreak response activities at the level of pre-certification and certification unit costs in endemic/pre-certified and certified countries, respectively, including multi-country activities. A high degree of uncertainty about the costs of both the GWEP and control scenarios means that these estimates overlap.

Our estimate of the cost of the GWEP in the period 1986–2020 is, in undiscounted nominal dollars, US$ 432 million (95% uncertainty interval, US$ 351–553 million). Our estimate for the period 1986–2004 is US$ 182 million (US$ 117–280 million), higher than but not inconsistent with the US$ 125 million reported elsewhere.[8]

### Markov model

**Fig 5:**
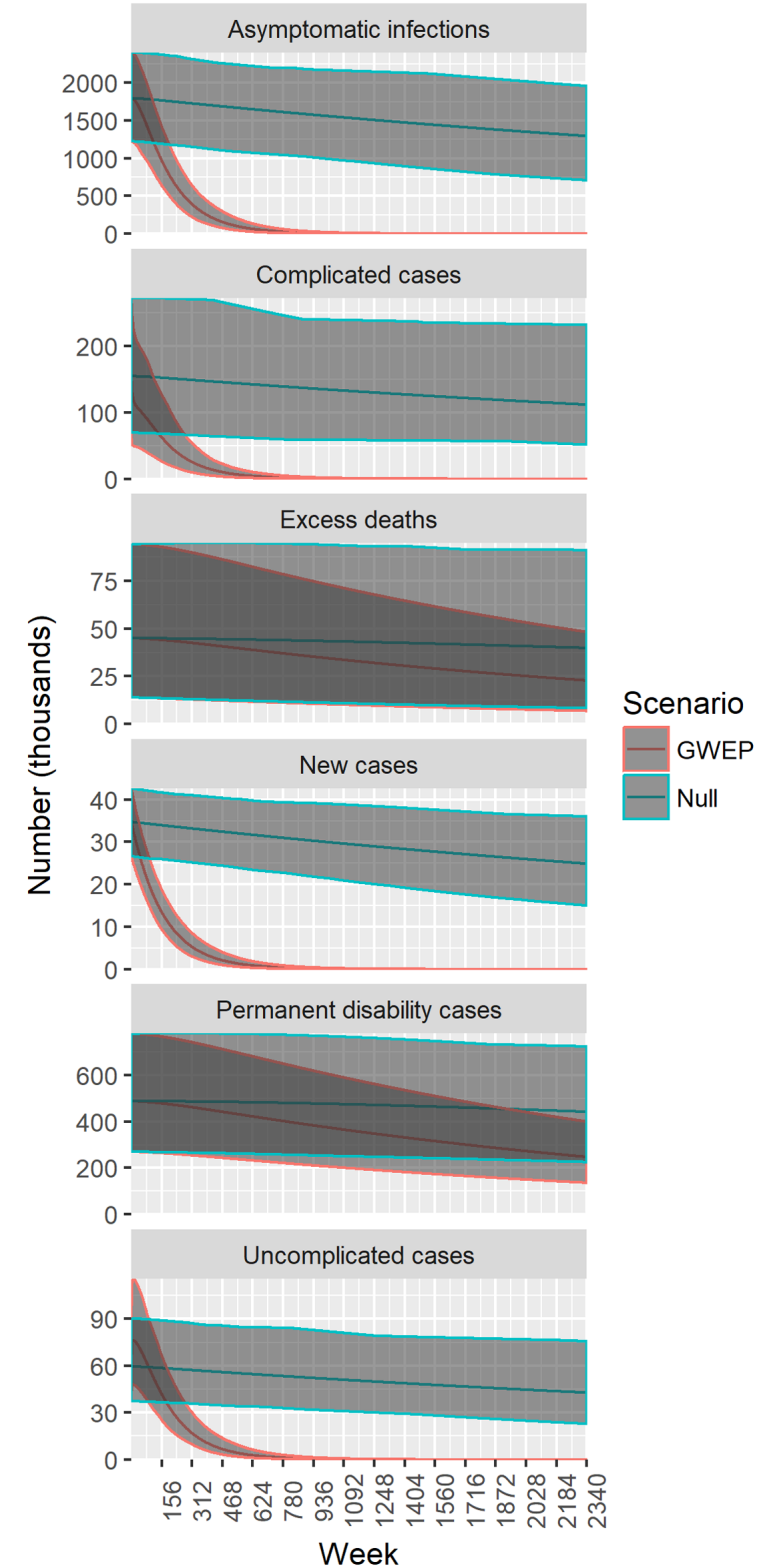
Estimated weekly number of infections, cases and deaths—GWEP and null scenarios, 1986–2030. The null scenario assumes no intervention since 1986.

In Fig 5 we present the results of two runs of the Markov model over a period of 2340 weeks (1986–2030): estimated weekly number of new cases, prevalent infections and disabilities and excess deaths under the GWEP and null scenarios. With 0.985–0.999 secondary cases per index case, the number of new cases decreases slowly under the null scenario. With an effective reproduction number of 0.59–0.74, the number of new cases drops rapidly under the GWEP, driving the decrease in the prevalence of asymptomatic infections, uncomplicated cases, complicated cases and permanent disabilities. The number of excess deaths is also lower under the GWEP, but the effect is small and uncertain.

**Fig 6:**
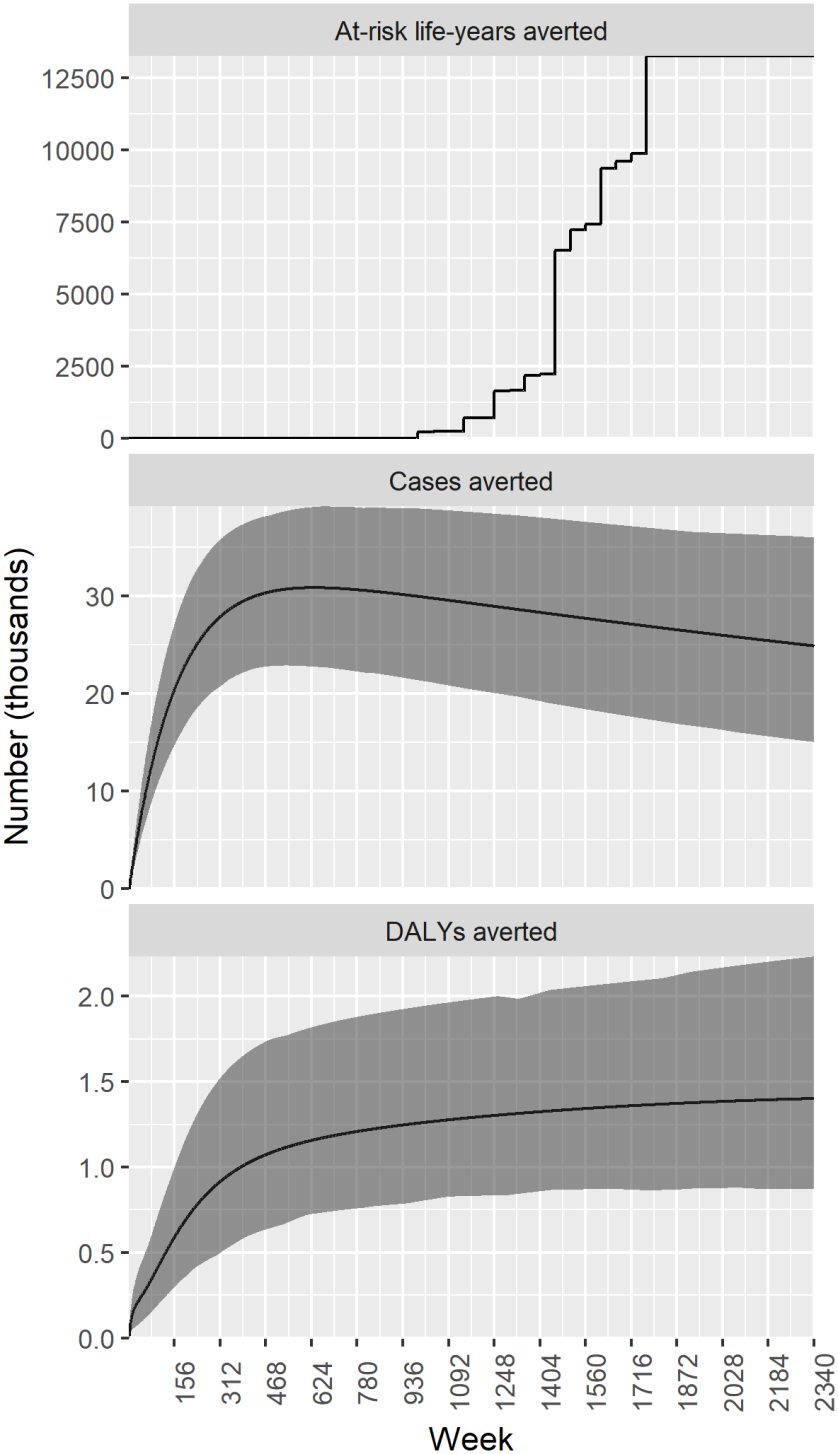
Estimated weekly number of at-risk life years, cases and DALYs averted by the GWEP relative to the null scenario, 1986–2030.

Fig 6 shows the estimated weekly number of at-risk life years, cases and DALYs averted by the GWEP relative to the null scenario, again over the period of 2340 weeks (1986–2030). The vast majority of cases are averted early on in the GWEP. About 25–30 thousand cases are averted each week, or 1.3–1.6 million cases per year. The total number of DALYs averted increases more gradually over the period 1986–2030. In terms of at-risk life years averted, impact is much more recent, with large numbers of people removed from risk of GWD in 2013 and after 2015.

### Cost-effectiveness

Table 2 gives the average cost-effectiveness ratios, with best estimates and 95% uncertainty intervals, for the GWEP compared to the null scenario. In its first decade (1986–1996), the GWEP is estimated to have cost about US$ 34 per case averted. By 2020, it cost about US$ 11 per case averted. This result is more conservative than that obtained by the WB study, which put the cost per case averted at about US$ 10 (or US$ 5 at 1987 prices) in 1986–1998; their reported result did not include time discounting of cases averted or allow for a decreasing number of cases in the absence of the GWEP. In the period 1986–2030, the cost per DALY averted by the GWEP relative to doing nothing is estimated at US$ 222 (118–372), much less than US$ 500 or one half of GDP per capita in most low-income countries. The cost per at-risk life year averted is much lower, at about US$ 0.06.

**Table 2:** Average cost-effectiveness ratio, 2015 US$, best estimate and 95% uncertainty intervals.

GWEP versus null^1^			
Period 1986-	Cost per at-risk life year averted	Cost per case averted	Cost per DALY averted
1996	NA^2^	33.83 (21.06–50.61)	1081 (557–1891)
2020	0.17 (0.12–0.25)	10.94 (6.62–16.80)	280 (150–475)
2030	0.06 (0.04–0.09)	9.18 (5.42–14.45)	222 (118–372)

^1^ The null is the do nothing scenario, with zero costs and a natural history of disease since 1986.

^2^ No at-risk life years averted, since the first GWD endemic country was certified in 2005.

**Fig 7:**
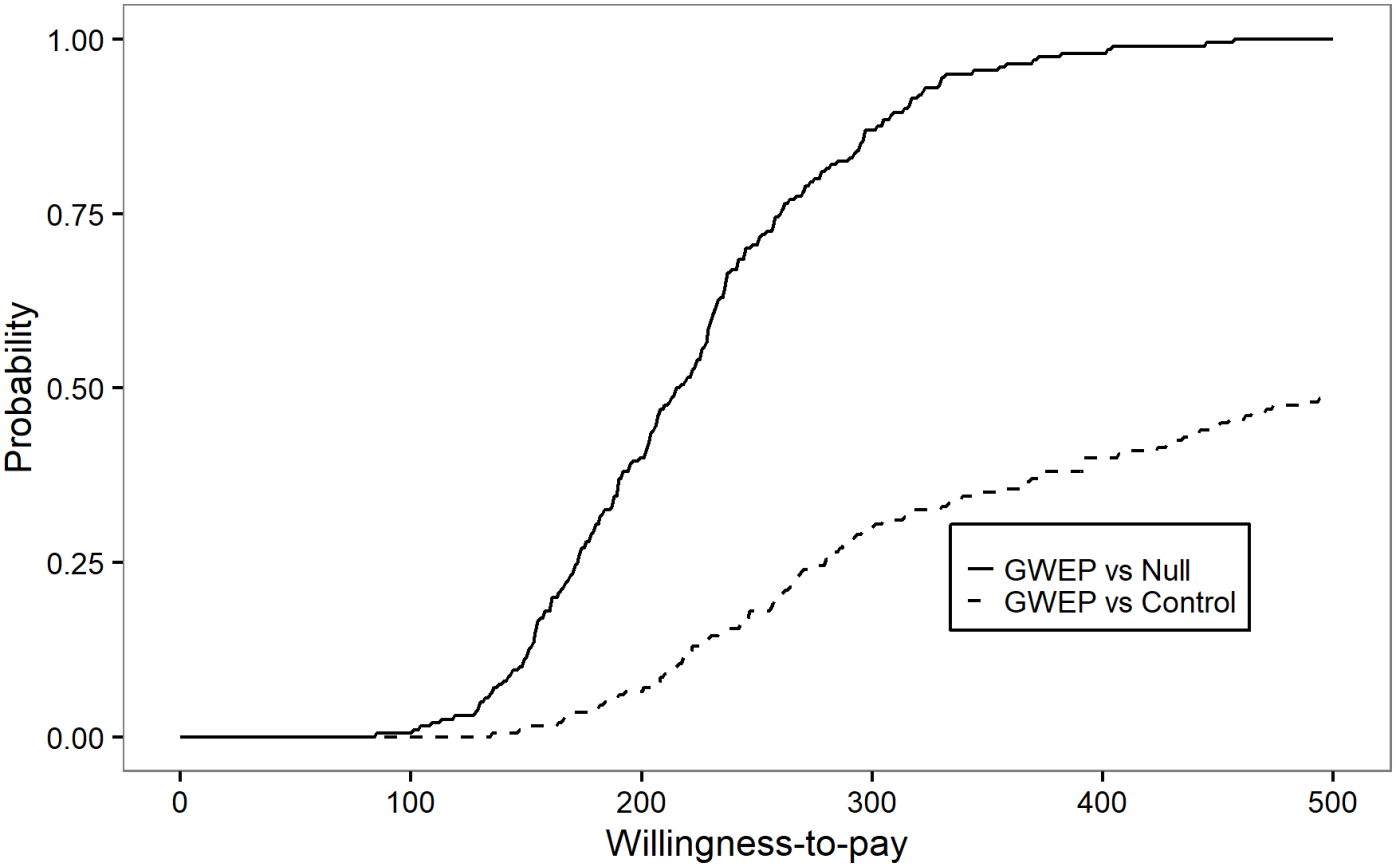
Probability of being cost-effective, by willingness-to-pay (US$) for a DALY averted, in the period 1986–2030. The control scenario assumes surveillance and outbreak response activities at the level of pre-certification and certification unit costs in endemic/pre-certified and certified countries, respectively, including multi-country activities; it further assumes that these surveillance and outbreak response activities succeed in maintaining incidence at 2015 levels.

Fig 7 shows cost-effectiveness acceptability curves (CEACs) for the comparison of the GWEP to both the null (do nothing) and control scenarios. The CEAC represents the probability that an intervention is cost-effective across a range of possible thresholds of willingness-to-pay. Values of the CEAC closer to 1 indicate that uncertainty in the cost-effectiveness of the reference intervention is very low.[22] Fig 7 (solid line) shows that the probability that the GWEP is more cost-effective than doing nothing exceeds 90% at a willingness-to-pay threshold of about US$ 300 per DALY averted.

Whether the GWEP is more cost-effective than the control scenario is less clear in terms of the cost per DALY averted, given uncertainty about the costs of both scenarios (Fig 7, dashed line). Recall that we have assumed that surveillance and outbreak response activities at the level of pre-certification and certification unit costs in endemic/pre-certified and certified countries, respectively, succeed in maintaining incidence at 2015 levels. This assumption puts the probability of cost-effectiveness of GWEP compared to control at nearly 50% (a coin toss), at a willingness to pay threshold of about US$ 500 per DALY averted.

**Fig 8:**
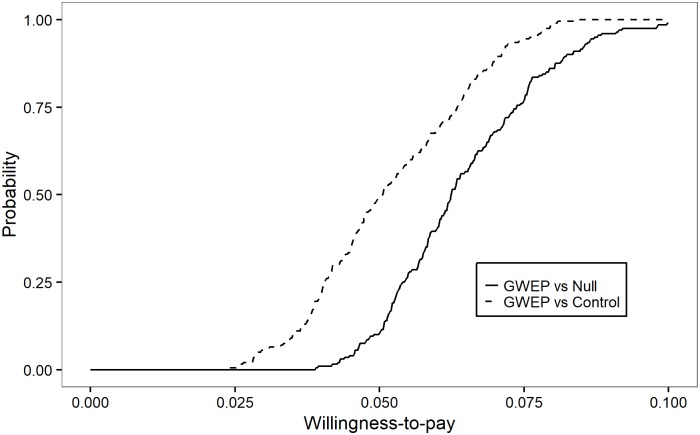
Probability of being cost-effective, by willingness-to-pay (US$) for an at-risk life year averted, in the period 1986–2030. The control scenario assumes surveillance and outbreak response activities at the level of pre-certification and certification unit costs in endemic/pre-certified and certified countries, respectively, including multi-country activities; it further assumes that these surveillance and outbreak response activities succeed in maintaining incidence at 2015 levels.

Fig 8 is another CEAC, similar to Fig 7, but in which the willingness-to-pay thresholds are expressed per at-risk life year averted. It shows that the GWEP is certainly more cost-effective than both the do nothing and control scenarios, if willingness to pay for one year of life lived without the risk of GWD exceeds US$ 0.10.

## Discussion

The GWEP continues to be highly cost-effective in the period 1986–2030. Even if economic costs are two times as high as the financial costs estimated for the period to 2020, the GWEP will still be cost-effective relative to doing nothing.

Whether the GWEP turns out to more cost-effective than a simple control strategy in the period beyond 2015 will depend much on the time horizon. The longer the time horizon, the greater the cost of control relative to the GWEP (assuming eradication is indeed achieved by 2020).

The benefits of eradication may not be fully captured by standard metrics such as cost per DALY averted. When framed in terms of the number of years of life lived without the risk of GWD, a case can be made more easily for finishing the end game, including certification of the absence of transmission. We refer the reader to an extensive review of all the health and economic benefits that can be attributed to a “year of life lived without the risk of GWD”.[23]

To the best of our knowledge, ours is one of the first analyses of the cost-effectiveness of an eradication programme in the end game. Most economic evaluations of eradication and elimination programmes do not explicitly break down the costs over time. Among the few studies that provide costs over time, the vast majority do not break by category or phase. Only one study (of polio eradication) considered the cost of certification, estimated at US$ 492 million in the post-elimination period.[24]

Another study examined the difference in costs between control and eradication of polio.[25] They found that although control has a lower initial annual cost, the cumulative costs of the two strategies will become equal after 6 years and thereafter the control strategy will cost more. Moreover after 20 years, the control strategy will cost US$ 800 million more than the eradication strategy.

In addition to more up-to-date and comprehensive data, this study has several strengths relative to a 1997 evaluation of the GWEP by the WB. We have developed a Markov model to estimate the number of DALYs averted. We have performed probabilistic sensitivity analysis on the most uncertain parameters.

Our study does have limitations. First, we have not considered all possible alternatives to the GWEP as it was implemented.

In theory, accelerated scale up might have involved higher annual costs over lower number of years for some of the endemic countries. In practice, however, the end point of global eradication is unlikely to have come much sooner. The primary constraint to scale up has been security concerns preventing full access to endemic areas—sometimes for months or years. As a matter of fact, all the four remaining endemic countries have experienced serious security concerns. Sudan and South Sudan have experienced uninterrupted conflict since the start of the GWEP.

Also in theory, scale up might have been faster or cheaper if some of the cost of global and regional level activities had been shifted to the country level. In practice, however, without global and regional coordination, the GWEP would not have been successful in mobilizing the necessary resources, including financial, human as well as political capital. Without independent verification that countries had met all the criteria for being certified free of the disease, global eradication would not be possible.

A less theoretical limitation is that not all costs have been included. We have not included, for example, the cost to UNICEF of providing boreholes. Note, that we have also not included the collateral benefits of these public health goods on other diseases. Some smaller donors have supplemented intervention costs (for example, the Japan International Cooperation Agency in Ghana).

The GWEP has benefitted from volunteers and general staff of national Ministries of Health and local non-governmental organizations. In some countries, small rewards have been paid by Ministries of Health directly. We were unable to survey national GWEPs to obtain data on ministry of health staff time used for the purposes of implementing programmes. To our knowledge, the WB study also did not include these domestic contributions.

Furthermore, projected costs in 2016–2020 might be too low. The budget envisaged in this analysis is for US$ 111 million. TCC originally developed a proposal for $210 million, with the last case in 2016 and global certification by 2020.

On the other hand, we have certainly omitted some of the benefits, including DALYs averted. A household survey in Sudan found that “children were three times more likely to be malnourished if more than half the adult members had suffered from the GWD in the previous year.”[26] In countries with ongoing activities, the national GWEP maintains surveillance for both GWD and acute flaccid paralysis (for polio surveillance).

Some of the financial costs included are not incremental costs, but rather costs shifted from the national health system and patients to the GWEP. In South Sudan, Ethiopia and Mali the vast majority if not all patients are hospitalized at GWEP case containment centres. Only in Chad have patients, so far, been hospitalized at health units or district hospitals serving the affected areas. Included in the TCC costs is between $100 and $200 per patient for first aid treatment costs associated with GWD, including occlusive bandages, topical antibiotic, a bed mat, bed sheets, a mosquito net and three meals a day.

Like the WB study, we have ignored “the benefits in terms of reduced infection-related expenditures among cases as well as positive effects on savings and income in the long run”.[7] The GWEP is thought to help keep children in school by stopping children getting the disease and preventing their parents becoming unwell, requiring their children to work in their place.[27]

Nor have we amortized the financial cost of assets over their useful lives, including the case containment centers built in South Sudan, Ethiopia and Mali. The legacy of GWEP’s established health infrastructure and networks will include community-based surveillance and health education delivery systems that are poised to deliver other essential interventions. The system of trained village volunteers undertaking health promotion and surveillance are already used for other health activities.

All these limitations considered, even if the true economic cost of eradication were as much as two times higher than the costs we have estimated for the period 1986–2030, the GWEP would still be cost-effective relative to the alternative that was decisively rejected by TCC, WHO and their partners in 1986 –doing nothing. Having re-asserted the cost-effectiveness of the GWEP as a whole, future research could help identify which of its individual components have driven that cost-effectiveness, and whether benefits could have been delivered earlier, or at lower cost.

## Supporting information

S1 Table Risk-based surveillance for Guinea Worm Disease Eradication. (DOCX)

S2 Table Epidemiological parameters for the Markov model of guinea worm disease. (DOCX)

S1 Fig Markov model of guinea worm disease. Epidemiological parameters (E1-E11) are described in S2 Table.
(PNG)
