# Supplementary material for: The cost-effectiveness of an eradication programme in the end game: Evidence from guinea worm disease
Source: PLoS Negl Trop Dis. 2017 Oct 5;11(10):e0005922. doi: 10.1371/journal.pntd.0005922 (PMC5628789; doi:10.1371/journal.pntd.0005922)
Supplement: S1 Table — (DOCX) [file pntd.0005922.s001.docx]

| **Risk level** | **Risk factors** | **Priority surveillance tasks** | **Targets** |
| --- | --- | --- | --- |
| **Endemic** | - Endemic districts / villages | 1. *Daily* active community-based surveillance in all endemic districts and high risk localities (refugee camps, border communities, etc.) 2. Daily supervision of health workers and community volunteers 3. Cash reward awareness, health education and self-reporting using additional inter-personal cost-effective channels (including town criers) 4. Monthly training /re-orientation of health workers and community volunteers 5. Ensure province and district surveillance logistics 6. Ensure provincial and district focal person to support surveillance | 1. 100% villages placed under active surveillance 2. Monthly reporting from all villages under active surveillance 3. > 85% of health facility report monthly, including zero cases report 4. 100% of districts report monthly, including zero cases report 5. Awareness on cash reward among general population at least 70% 6. 100% rumours investigated within 24hrs and hospitalized for observation 7. 100% districts should maintain a list of all village volunteers and supervisors with their mobile phone numbers, if available. |
| **High risk** | - Recently freed districts/zones - Districts/zones hosting refugees from endemic districts - Districts with cross-border population movements with endemic or recently freed districts/zones | 1. Additional intensified cash reward awareness, health education and self-reporting using additional inter-personal cost-effective channels 2. *Frequent active* community-based surveillance in all high-risk villages 3. Quarterly case searches in high risk localities, refugee camps and border communities 4. Quarterly training of health workers and community volunteers 5. *Monthly* supervision of health workers and community volunteers 6. Quarterly mapping of refugee camps (and other high-risk populations), including inventories of health infrastructure. 7. Maintain volunteers and emergency response teams in camps and other high-risk localities 8. Ensure district surveillance logistics and focal person to support surveillance 9. Each country should have a plan to ensure that surveillance and interventions continue when conflicts and insecurities lead to difficulty in accessing the villages from the district or higher levels. 10. Create a network of surveillance staff by hiring and training/briefing locally recruited persons (tailored to local context and individual ethnic group sensibility) and providing them with financial incentive and logistics (cell phones, “air time”, speakers, motorbikes) to disseminate message on the cash reward as well ensure exhaustive rumour notification/investigation within 24 hours. 11. For investigation of suspected cases and implementation of interventions, access could be enabled through United Nations (UN) Department of Safety and Security and humanitarian agencies both from the UN system as well as NGOs 12. NGOs with field presence in these areas should be tapped in to enhance the surveillance network. 13. In areas of insecurity, supervision and cross-checking of such surveillance will be conducted remotely primarily by means of telephone or VHF/UHF radio. | 1. 100% villages placed under active surveillance 2. At least 85% of all villages under active surveillance should report on a monthly basis for at least 9 months in a year 3. 100% of all villages with active cases should report every month. 4. > 85% of health facility report monthly, including zero cases report 5. 100% of districts report monthly, including zero cases report 6. Awareness on cash reward among general population at least 70% 7. 100% rumours investigated within 24hrs 8. 100% districts should maintain a list of all village volunteers and supervisors with their mobile phone numbers, if available. 9. and high-risk villages have rumour records/registers available |
| **Normal risk** | - Never endemic districts - Formerly endemic districts which have completed the mandatory 3 year period of active surveillance (with no report of cases and no risk of movement of population to/from endemic areas) | 1. Cash reward awareness, health education and self-reporting through public media (radio & TV) 2. Annual / ad hoc awareness surveys 3. *Passive* surveillance through Integrated Disease Surveillance and Response (IDSR) or other existing system 4. Annual / ad hoc case searches – integrated with community-based health activities (e.g. national immunization day, mass drug administration campaigns, etc.) 5. Supervision at least quarterly 6. NGOs with field presence in these areas should be tapped in to enhance the surveillance network. 7. Supervision and information through remote access by mobile phone, radio network | 1. 85% of health facility report monthly, even if zero cases 2. 100% of districts report monthly, even if zero cases 3. Awareness on cash reward among general population at least 50% 4. 100% rumours investigated within 24hrs 5. Rumour records available in all districts and State/Regional offices |
| **All Risk Levels**  National Eradication Awareness Day | | | |
