# Supplementary material for: The cost-effectiveness of an eradication programme in the end game: Evidence from guinea worm disease
Source: PLoS Negl Trop Dis. 2017 Oct 5;11(10):e0005922. doi: 10.1371/journal.pntd.0005922 (PMC5628789; doi:10.1371/journal.pntd.0005922)
Supplement: S2 Table — (DOCX) [file pntd.0005922.s002.docx]

|  | | Distribution | Parameters | | Sources | Note |
| --- | --- | --- | --- | --- | --- | --- |
|  |  |  | Counterfactual | GWEP |  |  |
| E1 | Reproduction number | Uniform | Min=0.985  Max=0.999 | Min=0.59  Max=0.74 | This study. | Conservative assumption of no increase in numbers; on the low end, we allow for 1.5% decline over the generation time. For the GWEP, we estimated the effective reproduction number using monthly data on the number of cases. |
| E2 | Generation time (weeks) | Uniform | Min= 45  Max=65  including 43-61 weeks of asymptomatic infection. | same | Cairncross, S., Muller, R., and Zagaria, N. (2002). Dracunculiasis (Guinea Worm Disease) and the Eradication Initiative. Clinical Microbiology Reviews *15*, 223–246. | From the time infection occurs, it takes between 10–14 months (43–61 weeks) for the transmission cycle to complete until a mature female worm emerges from the body.When submerged in water, the female worm releases larvae. Consumed by copepods, larvae develop to the infective third stage in 14 days (2 weeks); infected copepods live up to 4 weeks. |
| E3 | Duration of disease before any complications (weeks) | Uniform | Min=2  Max=4 | same | Aehyung Kim, Ajay Tandon and Erenesto Ruiz-Tiben (1997). Cost-benefit analysis of the global dracunculiasis eradication campaign (GDEC) | Symptoms persist a minimum of 2 weeks in the absence in any complications. |
| E4 | Disability weight without complications | Beta | α = 22.07927  β = 94.23169 | same | Disability weights for the Global Burden of Disease 2013 study, available at:  <http://www.thelancet.com/pdfs/journals/langlo/PIIS2214-109X(15)00069-8.pdf> | *Disfigurement: level 2*, *with itch or pain*; a visible physical deformity that is sore and itchy. Other people stare and comment, which causes the person to worry. The person has trouble sleeping and concentrating.  Mean: 0.188  95% CI: 0.125–0.267 |
| E5 | Probability of complications | Uniform | Min=0.50  Max=0.76 | Min=0.25  Max=0.50 | Cairncross, S., Muller, R., and Zagaria, N. (2002). Dracunculiasis (Guinea Worm Disease) and the Eradication Initiative. Clinical Microbiology Reviews *15*, 223–246. | Severe incapacitation is associated with secondary infection of the lesion; this occurs in roughly half of cases; early studies from Nigeria (1989-1991) suggest 58-76% of patients were unable to leave their beds. For the GWEP, we assumed that complications were as much as halved. |
| E6 | Duration of disease after any complications (weeks) | Tri-angular | Min=2  Mode=8  Max=16 | same | Aehyung Kim, Ajay Tandon and Erenesto Ruiz-Tiben (1997). Cost-benefit analysis of the global dracunculiasis eradication campaign (GDEC) | A review of twelve  studies suggests an average duration of disability of about 8 weeks (estimates ranging from 2 to 16 weeks). |
| E7 | Disability weight with complications | Beta | α = 20.51711  β = 48.57165 | same | Disability weights for the Global Burden of Disease 2013 study, available at:  <http://www.thelancet.com/pdfs/journals/langlo/PIIS2214-109X(15)00069-8.pdf> | *Gout: acute;* severe pain and swelling in the leg, making it very difficult to get up and  down, stand, walk, lift, and carry heavy things. The person has trouble  sleeping because of the pain.  Mean: 0.295  95% CI: 0.196–0.409 |
| E8 | Probability of permanent disability from GWD complications | Beta | α = 12.60608  β = 2442.91970 | same | Hours, M., and Cairncross, S. (1994). Long-term disability due to guinea worm disease. Trans. R. Soc. Trop. Med. Hyg. *88*, 559–560.  Cairncross, S., Muller, R., and Zagaria, N. (2002). Dracunculiasis (Guinea Worm Disease) and the Eradication Initiative. Clinical Microbiology Reviews *15*, 223–246. | The prevalence of permanent disability among 195 cases was 0.5% (95% CI 0.017–0.832%); a number of studies have found it to be less than 1%. |
| E9 | Disability weight for permanent disability | Beta | α=28.44683  β=328.09786 | same | Disability weights for the Global Burden of Disease 2013 study, available at:  <http://www.thelancet.com/pdfs/journals/langlo/PIIS2214-109X(15)00069-8.pdf> | *Musculoskeletal problems: legs, moderate;* moderate pain in the leg, which makes the person limp, and causes some  difficulty walking, standing, lifting and carrying heavy things, getting up  and down and sleeping.  Mean: 0.079  95% CI: 0.054–0.110 |
| E10 | Natural mortality | Deterministic | Country-specific | same | World Population Prospects: The 2017 Revision, using life expectancy at age 1, both sexes combined, in the period 2010-2015, available at:  <https://esa.un.org/unpd/wpp/Download/Standard/Mortality/> | Life expectancy is a country-specific value. |
| E11 | Case fatality rate from GWD complications | Tri-angular | Min=natural mortality*1.01  Mode=0. 1%  Max= 0.3% | same | Cairncross, S., Muller, R., and Zagaria, N. (2002). Dracunculiasis (Guinea Worm Disease) and the Eradication Initiative. Clinical Microbiology Reviews *15*, 223–246. | Studies in India based on medical records suggest a case fatality rate of 0.1%; a study from Benin suggests 0.3% from tetanus and septicaemia. |
