# Supplementary figures and images for: The cost-effectiveness of an eradication programme in the end game: Evidence from guinea worm disease
Source: PLoS Negl Trop Dis. 2017 Oct 5;11(10):e0005922. doi: 10.1371/journal.pntd.0005922 (PMC5628789; doi:10.1371/journal.pntd.0005922)

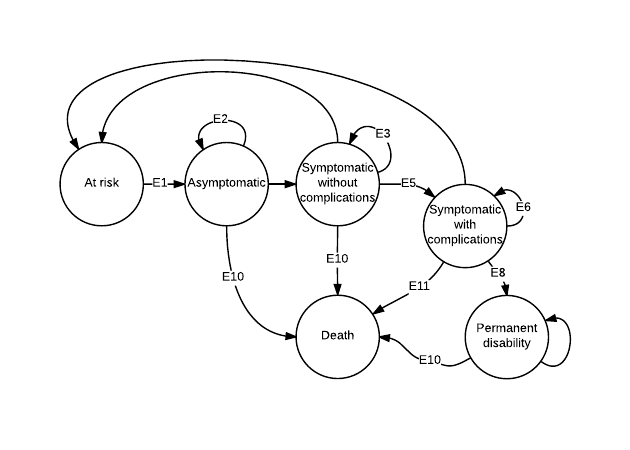

Supplement: S1 Fig — Epidemiological parameters (E1-E11) are described in S2 Table. (PNG) [file pntd.0005922.s003.png]
